# The Effect of Cachexia on the Feeding Regulation of Skeletal Muscle Protein Synthesis in Tumour‐Bearing Mice

**DOI:** 10.1002/jcsm.70064

**Published:** 2025-09-10

**Authors:** Brittany R. Counts, Quan Zhang, Jessica L. Halle, Melissa J. Puppa, Stephen E. Alway, Junaith Mohamed, Jeremy P. Loenneke, James A. Carson

**Affiliations:** ^1^ Integrative Muscle Biology Laboratory, Division of Rehabilitation Sciences, College of Health Professions University of Tennessee Health Science Center Memphis Tennessee USA; ^2^ Department of Cell, Development and Cancer Biology, Knight Cancer Institute Oregon Health & Science University Portland Oregon USA; ^3^ Integrative Muscle Biology Lab, Department of Kinesiology & Sport Management Texas A&M University College Station Texas USA; ^4^ Oklahoma Medical Research Foundation Oklahoma City Oklahoma USA; ^5^ School of Health Studies University of Memphis Memphis Tennessee USA; ^6^ Laboratory of Muscle Biology and Sarcopenia, Department of Physical Therapy, College of Health Professions University of Tennessee Health Science Center Memphis Tennessee USA; ^7^ Laboratory of Nerve and Muscle, Department of Diagnostic and Health Sciences, College of Health Professions University of Tennessee Health Science Center Memphis Tennessee USA; ^8^ Department of Health, Exercise Science, and Recreation Management, Kevser Ermin Applied Physiology Laboratory The University of Mississippi University Mississippi USA; ^9^ Huffines Institute for Sports Medicine & Human Performance Texas A&M University College Station Texas USA

**Keywords:** anabolism, cancer, fasting, feeding, inflammation

## Abstract

**Background:**

Cancer promotes muscle wasting through an imbalance in the tightly regulated protein synthesis and degradation processes. An array of intracellular signalling pathways, including mTORC1 and AMPK, regulate protein synthesis, and these pathways are responsive to the muscle's microenvironment and systemic stimuli. Although feeding and fasting are established systemic regulators of muscle mTORC1 and protein synthesis, the cancer environment's impact on these responses during cachexia development is poorly understood. Although the IL‐6 cytokine family has been widely investigated as a driver of cachexia with several cancers, how this signalling regulates muscle responses to feeding and fasting requires further study. We investigated if the cancer environment alters the feeding and fasting regulation of skeletal muscle protein synthesis and if the IL‐6 family of cytokines signalling through muscle glycoprotein 130 could regulate this response.

**Methods:**

Male C57BL/6J mice were subcutaneously injected with 1 × 10^6^ LLC cells or PBS. Mice were euthanized 25–30 days post‐injection after a 12‐h dark cycle fast, followed by access to food pellets for 1 h (fed) or immediately sacrificed. To determine AMPK and gp13's regulation of protein synthesis and anabolic signalling, we injected tamoxifen‐inducible skeletal muscle AMPKa^**1**^a^**2**^ or gp130 knockout and floxed control mice with LLC cells or PBS. The gastrocnemius muscle was analysed for protein expression.

**Results:**

Feeding increased p‐rpS6 and protein synthesis in PBS (2.2‐ and 0.4‐fold, *p* < 0.001) and LLC mice (1.7‐ and 0.9‐fold, *p* < 0.001), but overall, LLC significantly reduced p‐rpS6 and protein synthesis. Feeding only increased p‐AKT in PBS mice (1.5‐fold, *p* < 0.001). In vitro LLC‐conditioned media did not inhibit the insulin induction of myotube p‐AKT (*p* < 0.001) and p‐rpS6 (*p* < 0.001). Muscle gp130 loss reduced the fasting p‐AMPK induction in LLC mice but did not alter suppression of p‐AKT and p‐rpS6 and protein synthesis. Muscle AMPK loss increased p‐rpS6 (2.1‐fold, *p* < 0.001) and protein synthesis (0.7‐fold, *p* < 0.001) in PBS mice but did not restore LLC‐suppressed protein synthesis.

**Conclusions:**

Our study provides novel insight into muscle responsiveness to feeding and fasting in a cancer environment. We find the acute anabolic response to feeding is maintained during LLC‐induced cachexia, whereas the fasting catabolic response is exacerbated. Muscle‐specific gp130 loss prevented disrupted fasting AMPK activation but not protein synthesis. There is a need to understand the aberrant upstream and downstream regulation of muscle AMPK activity that is disrupted with cancer and leads to aberrant protein turnover regulation.

## 1 Introduction

Lung cancer has the second‐highest diagnosis rate in the United States, and 50% of patients experience cachexia [1]. The loss of muscle with cancer cachexia is linked to impaired chemotherapy tolerance, physical dysfunction and reduced cancer patients' survival and quality of life [2]. Systemically, cancer can drive cachexia by a combination of factors, including reduced food intake, metabolic dysfunction, elevated energy expenditure and increased systemic inflammation [3]. The response of skeletal muscle to these cancer‐induced systemic alterations is fundamental to regulating muscle protein turnover, which is required for maintaining muscle mass. Systemic and tissue metabolic flexibility is critical for maintaining health and demonstrates the importance of dynamic regulatory responses to fasted and fed conditions and increased or decreased physical activity [4]. Disrupted flexibility leads to metabolic dysfunction and poor health outcomes. Whole‐body metabolic homeostasis has been reported to be disrupted with cachexia in several preclinical models, including the Lewis Lung Carcinoma (LLC) model [5]. Furthermore, lipid metabolism is accentuated in LLC mice progressing to cachexia [6]. However, there is a limited mechanistic understanding of skeletal muscle's responsiveness to feeding and fasting in preclinical cancer cachexia models, including the LLC model. Beyond measuring food consumed, this lack of knowledge is a limitation for determining how feeding and fasting can impact muscle anabolic and catabolic processes that drive cancer‐induced muscle wasting. As there are limited options to successfully prevent or attenuate cancer cachexia's progression, an improved mechanistic understanding of skeletal muscle metabolic flexibility will provide foundational knowledge for more effective behavioural, nutritional and pharmaceutical strategies to treat or attenuate cachexia and improve cancer patients' quality of life and survival.

The mechanistic target of rapamycin complex 1 (mTORC1) and 5′ adenosine monophosphate‐activated protein kinase (AMPK) signalling have regulatory roles in skeletal muscle protein turnover responses to local and systemic stimuli [7, 8], including the responses to feeding and fasting conditions [9]. Importantly, the cancer patient's anabolic response to feeding has been reported to be suppressed [10], including muscle protein synthesis [11]. Basal protein turnover dysregulation has been extensively investigated in preclinical cancer cachexia models [12]. Cancer‐suppressed anabolic signalling can involve aberrant mTORC1 and AMPK activation [7, 13]. In a preclinical colon cancer model, we have reported that diurnal muscle mTORC1 signalling related to feeding and cage activity is disrupted [14]. The skeletal muscle intracellular anabolic response to feeding in healthy conditions is well established [15], and acute feeding can induce skeletal muscle mTORC1, suppress AMPK activity [16] and increase protein synthesis [9]. However, there is a limited mechanistic understanding of how cancer disrupts skeletal muscle mTORC1 and AMPK signalling responses to feeding and fasting stimuli.

Systemic inflammation and associated skeletal muscle signalling have been extensively studied as drivers of muscle wasting in cancer patients and preclinical cancer models [17, 18]. The interleukin‐6 (IL‐6) family of cytokines has been reported to regulate cachexia in several preclinical cancer models [19]. Signalling through the ubiquitously expressed transmembrane receptor glycoprotein‐130 (gp130) upon complex formation with specific IL‐6 family cytokine receptors activates intracellular signalling pathways, including MAPK and STAT3 [18, 19]. Additional downstream effectors interacting with this pathway can regulate muscle protein turnover, including NFkB [20] and processes involving ribosome biogenesis, protein synthesis, autophagy and ubiquitin‐proteasome degradation [21]. IL‐6/gp130 signalling can also phosphorylate AMPK independent of STAT3 activity [22]. Systemic IL‐6 overexpression in tumour‐bearing mice can accelerate muscle wasting [23], and high circulating IL‐6 levels coincided with suppressed muscle mTORC1 and upregulated AMPK signalling [12]. We have reported that muscle‐specific gp130 deletion in LLC tumour–bearing mice can attenuate muscle wasting but not rescue disrupted basal muscle AMPK and mTORC1 signalling [24]. A better mechanistic understanding of the role of gp130 signalling in the cancer disruption to AMPK and AKT/mTORC1 signalling and the impact on muscle metabolic flexibility is needed [13].

The rigour of the prior research investigating the cancer regulation of skeletal muscle protein turnover in preclinical cachexia models has been impacted by limitations in defining the fasting paradigm used to determine basal regulation or neglecting to examine the muscle responsiveness to feeding [15]. These issues have likely contributed to inconsistencies in the published literature involving the role of cytokine signalling in skeletal muscle protein turnover regulation, as the cancer systemic environment interacts with fasting and feeding periods throughout the daily cycle. Understanding the cancer systemic environment and inflammatory signalling impact on muscle AMPK and mTORC1 signalling during acute feeding and prolonged fasting could provide crucial insight into their roles in cachexia. Therefore, we investigated if the cancer environment could alter the feeding and fasting regulation of skeletal muscle protein synthesis and if the IL‐6 family of cytokines signalling through muscle gp130 protein regulated these processes. To this end, we used the established LLC preclinical cancer cachexia model to examine the anabolic muscle response to either acute feeding or a defined prolonged fast. Furthermore, muscle wasting was examined following inducible targeted muscle deletion of AMPK and gp130 in LLC and control mice.

## 2 Methods

### 2.1 Cell Culture

All cells were purchased through ATCC (Manassas, VA), and cell culture experiments were detailed in the Supporting Information. In brief, LLC cells were cultured for conditioned medium and inoculation. Murine C2C12 myoblasts were cultured for in vitro experiments as previously described [25, 26].

### 2.2 Animals

The generation of skeletal muscle‐specific knockout of gp130 and AMPKa1a2 mice is detailed in the Supporting Information. C57Bl/6J (B6), *Gp130* ^*fl/fl*^, gp130mKO, AMPKa_*1*_^*fl/fl*^a_*2*_^*fl/fl*^ and AMPKmKO male mice were kept under a 12‐h light:12‐h dark cycle and were given rodent chow ad libitum (Harlan Teklad Rodent Diet, no. 8604). Between 11 and 12 weeks of age (B6) or 13–14 weeks of age (WT and mKO), mice were injected with either phosphate‐buffered saline (PBS) or 1 × 10^6^ LLC cells subcutaneously in the right flank under isoflurane anaesthesia. All mice were fasted for 12 h during the dark cycle. At the start of the light cycle, mice were either given ad libitum for 1 h (fed) or sacrificed at the end of the 12 h fast (fast). Mice were anaesthetised under isoflurane, and ~500 μL of retro‐orbital blood was collected. Then, mice were euthanized by cervical dislocation, and tissues were rapidly dissected. Plasma and frozen muscles were stored at −80°C until analysis. Animal experiments were approved by the University of Tennessee Health Science Center's Institutional Animal Care and Use Committee.

### 2.3 Blood Glucose and Plasma Insulin and IL‐6

Blood glucose was determined using a standard and readily available glucometer (Contour Next, NJ) through tail snipping. Plasma insulin and IL‐6 concentrations were determined using enzyme immunoassay kits according to the manufacturer's instructions (Mercodia, no. 10‐1247 and ThermoFisher, no. KMC0061).

### 2.4 Protein Synthesis Measurement

As previously described, the Surface Sensing of Translation (SUnSET) technique was used to determine estimated muscle protein synthesis rates [26]. Briefly, puromycin (EMD Millipore, no. MABE343) was dissolved in sterile saline and delivered by intraperitoneal injection (0.04‐μmol/g body weight) 30 min before euthanasia. Puromycin was incorporated into newly synthesized peptides, reflecting estimated global protein synthesis rates, and was analysed by Western blot.

### 2.5 Western Blot

Western blot analysis was performed as previously described [14]. Briefly, the gastrocnemius muscle was homogenized in lysis buffer, and protein concentration was determined using the Bradford method. Crude gastrocnemius muscle homogenates were fractionated on 8%–12% SDS‐polyacrylamide gels and transferred to PVDF membranes. Membranes were stained with Ponceau red to verify equal loading and transfer. Membranes were then blocked at room temperature for 1 h in 5% non‐fat milk or 5% BSA‐Tris‐buffered saline with 0.1% Tween‐20 (TBST). The membranes were incubated in commercially available phosphorylated and total protein primary antibodies, which are detailed in Table S3. Briefly, primary antibodies were incubated overnight in 5% TBST milk or 5% TBST BSA. Membranes were then incubated in 5% milk‐TBST containing anti‐rabbit or anti‐mouse IgG horseradish‐peroxidase conjugated secondary antibody purchased from Cell Signalling for 1 h at room temperature. Enhanced chemiluminescence (ECL) (GE Healthcare Life Sciences, NJ) was used to visualize the antibody–antigen interactions. Immunoblot images were collected using a digital imager (Invitrogen iBright, MA) and quantified by densitometry using Image J software (NIH). The signals from phosphorylation antibodies were expressed relative to total protein on the same gel and quantified as phosphorylation to total ratio.

### 2.6 Statistical Analysis

Unpaired *t*‐test or two‐way ANOVA with Tukey's post hoc analysis when a significant interaction was present. The Spearman correlation coefficient was used to determine associations with cachexia progression and systemic metabolism. Values of *p* < 0.05 were considered significant.

## 3 Results

### 3.1 Feeding Does Not Affect the Cachectic Phenotype in LLC Tumour–Bearing Mice

**FIGURE 1:**
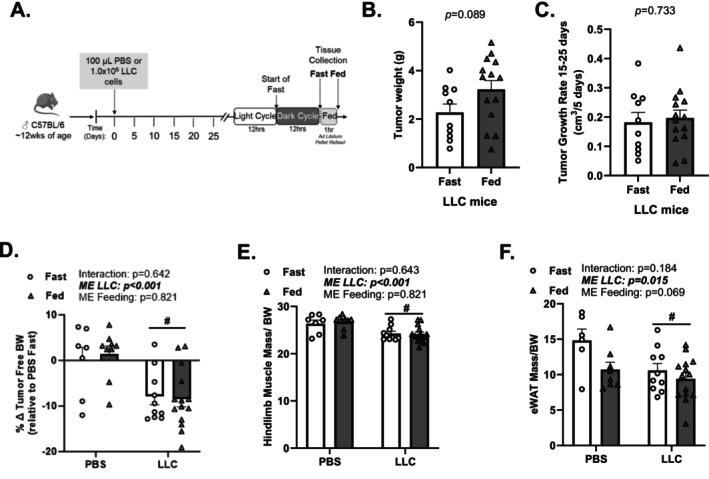
Effect of 1‐h ad libitum feeding on tumour burden and body composition in LLC tumour–bearing mice. (A) Study design. Male C57BL/6J mice were subcutaneously injected with 1 × 10^6^ LLC cells or PBS in the right flank at 12 weeks of age. After the 12‐h dark cycle, mice were euthanized after access to a food pellet for 1 h or fasted, 25–30 days post‐injection. (B) Tumour weight. (C) Tumour growth rate. (D) Tumour‐free final body weight normalized to PBS fast. (E) Hindlimb muscle mass includes gastrocnemius, soleus, plantaris, tibialis anterior and extensor digitorum longus to tumour‐free final body weight. (F) Epididymal white adipose tissue (eWAT) mass to tumour‐free final body weight. Data are presented as mean ± SEM Data are analysed using unpaired *t*‐test or two‐way ANOVA with Tukey's post hoc. Statistical significance was set to *p* < 0.05. *N* = 7–14 per group. #Main effect (ME) of LLC.

Male mice were injected with either LLC cells or an equivalent volume of PBS and euthanized in the fast or fed condition at 25–30 days after injection (Figure 1A). One hour feeding did not alter tumour burden or cachectic phenotype in LLC tumour–bearing mice. There was no difference in tumour weight or growth rate between the fast and fed LLC mice (Figure 1B,C). Body weight was not significantly different between PBS and LLC mice at the start and 10 days post‐LLC inoculation (Table 1). At the end of the experiment, LLC mice had a lower tumour‐free body weight (Figure 1D) and a greater tumour‐free body weight change from Day 10 to Day 0 compared with PBS mice (Table 1). In addition, LLC mice had a lower gastrocnemius mass (Table 1), hindlimb muscle mass (Figure 1E) and fat mass (Figure 1F). LLC mice had greater spleen weight and higher plasma IL‐6 primarily (Table 1). There was no difference in tibia length between PBS and LLC mice (Table 1). These data indicate that LLC mice demonstrated a cachectic phenotype determined by body weight and muscle mass loss.

**TABLE 1:** Effect of 1‐h ad libitum feeding on animal characteristics in LLC tumour–bearing mice.

	PBS		LLC		*p*		
	Fast	Fed	Fast	Fed			
*N*	7	9	10	14	Feeding × LLC	ME feeding	ME LLC
BW D 0 (g)	25.3 (1.0)	25.7 (0.6)	24.5 (0.6)	25.3 (0.4)	0.737	0.331	0.336
BW D10 (g)	26.5 (0.9)	26.5 (0.6)	26.1 (0.5)	25.4 (0.4)	0.554	0.600	0.225
Tumour‐free final BW (g)	26.4. (0.8)	26.7 (0.5)	24.5 (0.5)	24.2 (0.5)	0.640	0.825	***< 0.001***
BW change from D0 (%)	5 (2.4)	4.3 (1.7)	−0.3 (2.9)	−4.4 (1.8)	0.455	0.317	***0.004***
BW change from D10 (%)	−0.3 (1.1)	0.1 (1.1)	−6.8 (2.0)	−5 (1.7)	0.860	0.401	***< 0.001***
Gastrocnemius (mg)	117.8 (5.2)	117.1 (2.8)	111.1 (3.0)	105.2 (1.9)	0.394	0.284	***0.004***
HLM (mg)	192.9 (8.1)	190.5 (4.7)	180.6 (4.7)	170.9 (3.4)	0.463	0.239	***0.003***
eWAT (mg)	388 (45.6)	285.6 (28.0)	256.2 (22.5)	229.1 (22.4)	0.197	0.305	***0.002***
IL‐6 (pg/mL)	3.9 (0.0)	3.9 (0.0)	10.6 (2.6)	18.6 (3.0)	0.113	0.133	***< 0.001***
Spleen (g)	86.4 (7.8)	61.6 (5.1)	247.8 (61.8)	199.5 (14.4)	0.737	0.301	***< 0.001***
Tibia (mm)	17.0 (0.1)	16.7 (0.1)	16.9 (0.1)	16.7 (0.1)	0.514	0.071	0.707

*Note:* Data are presented as mean ± (SEM). Data are analysed using two‐way ANOVA with Tukey's multiple comparisons test. Statistical significance is set to *p* < 0.05. Bold and italicized text denotes a significant *p*‐value.

Abbreviations: BW: body weight; D0: day begin tumour inoculation; D10: Day 10 post–tumour inoculation; eWAT: epididymal white adipose tissue; g: grams; HLM: hindlimb muscle mass includes gastrocnemius, soleus, plantaris, tibialis anterior and extensor digitorum longus; LLC: Lewis Lung Carcinoma; mg: milligrams; mm: millimetres; PBS: phosphate‐buffered saline; pg/mL: picograms per millilitre.

### 3.2 Feeding Affects Systemic Glucose and Skeletal Muscle Protein Expression

**FIGURE 2:**
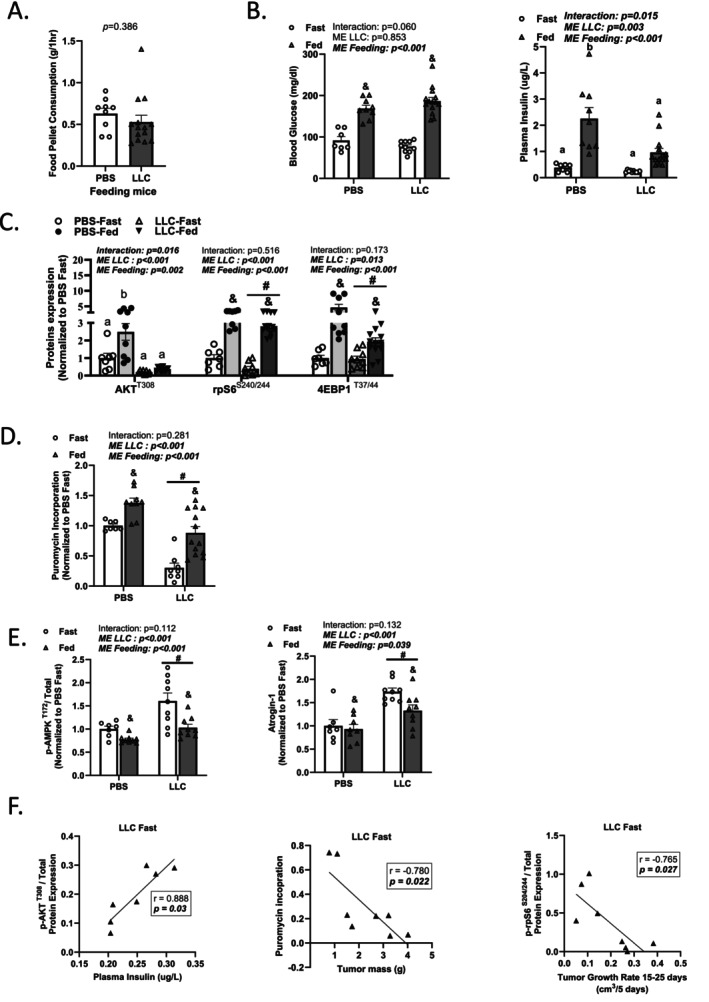
Systemic glucose and skeletal muscle response to feeding in LLC tumour–bearing mice. (A) Food pellet consumption. (B) Blood glucose and plasma insulin. (C) Quantified phosphorylated (p‐) to total AKT^(T308)^, rpS6^(S240/244)^ and 4EBP1^(T37/44)^ protein expression. (D) puromycin incorporation. (E) Quantified phosphorylated (p‐) to total AMPK^(T172)^ and atrogein‐1 protein expression. (F) Correlation of AKT with plasma insulin, puromycin incorporation with tumour mass and rpS6 with tumour growth rate. Data are presented as mean ± SEM. Data are analysed using unpaired *t*‐test, two‐way ANOVA with Tukey's post hoc and Pearson correlation. Statistical significance was set to *p* < 0.05. *N* = 7–14 per group. #Main effect (ME) of LLC. &Main effect (ME) of feeding. ‘a’ and ‘b’ mean significant difference between groups.

There was no difference in 1‐h food consumption between PBS and LLC‐fed mice (Figure 2A). Stomach mass and content increased in PBS and LLC‐fed mice compared with fasted mice (Table S1). Blood glucose increased in both PBS and LLC‐fed mice compared with fasted mice (Figure 2B). Although PBS‐fed mice increased plasma insulin compared with PBS fast, LLC‐fed mice exhibited suppressed insulin response compared with the PBS fed (Figure 2B).

We studied the AKT/mTORC1 and protein synthesis response to feeding (Figure 2C,D). As expected, feeding increased rpS6^(S240/244)^ and 4EBP1^(T37/44)^ phosphorylation and puromycin incorporation in PBS and LLC mice, whereas rpS6^(S240/244)^ phosphorylation and puromycin incorporation were lower in LLC compared with PBS mice. Although feeding increased AKT^(T308)^ phosphorylation in PBS mice, feeding did not induce AKT^(T308)^ phosphorylation in LLC mice. We examined AMPK signalling and have previously reported that AMPK signalling was induced in cachectic skeletal muscle after an overnight 12‐h fast in *Apc* ^*Min/+*^ mice [27]. Feeding reduced LLC‐induced AMPK^(T172)^ phosphorylation and atrogin‐1 expression (Figure 2E). These data support the mTORC1 and AMPK response to feeding being intact in cachectic LLC mice. The relationship between AKT/mTORC1, protein synthesis and AMPK with the cachectic phenotype, tumour development, glucose and IL6 in the LLC fast condition was investigated (Table S1 and Figure 2F). We report that AKT^(T308)^ is positively associated with plasma insulin levels in LLC fast mice and rpS6 was negatively associated with tumour growth rate. Puromycin incorporation was negatively associated with tumour mass, suggesting that tumour growth is linked to suppressed anabolic signalling. In conclusion, LLC mice exhibited a disrupted insulin response despite consuming similar food and a similar increase in blood glucose. In contrast, skeletal muscle mTORC1 and AMPK response to feeding remained intact.

### 3.3 LLC‐Conditioned Media Alters C2C12 Myotubes mTORC1 Response to Insulin and Leucine

**FIGURE 3:**
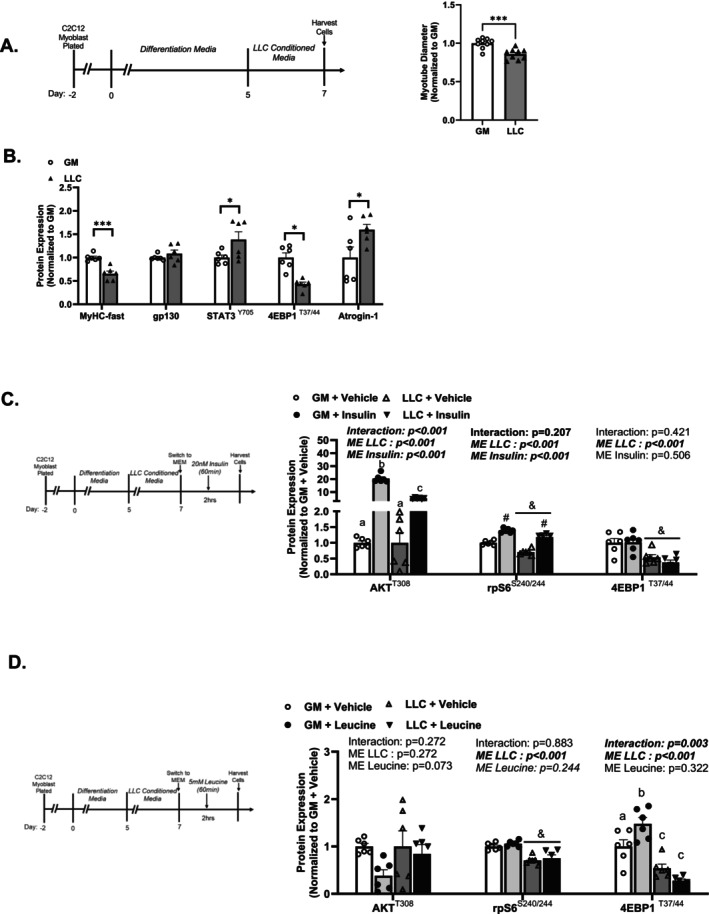
C2C12 myotubes treated with LLC conditioned media alter the mTORC1 response to insulin and leucine. (A) Experiment overview: C2C12 myoblasts are plated on Type I collagen‐coated plates in growth medium (GM, 10% FBS) and switched to differentiation medium (DM, 2% HS) after 48 h, which corresponds to Day 0 of differentiation. At Day 5 of differentiation, C2C12 myotubes were switched to high‐serum growth medium (GM, 5% FBS) or LLC medium for 48 h and myotube diameter. (B) MyHC‐fast, gp130, atrogin‐1 and quantified phosphorylated (p‐) to total STAT3^(Y705)^ and 4EBP1^(T37/44)^ protein expression. (C) Experiment overview: C2C12 myoblasts are plated on Type I collagen‐coated plates in GM (10% FBS) and switched to DM after 48 h, which corresponds to Day 0 of differentiation. At Day 5 of differentiation, C2C12 myotubes were switched to high‐serum growth medium (GM, 5% FBS) or LLC medium for 48 h. C2C12 myotubes were rinsed with PBS and switched to minimal essential medium (MEM) for 1 h on *Day 7* of differentiation and then treated with vehicle or 20‐nM insulin in MEM for 60 min. Representative Western blotting images with Ponceau S stain as a protein loading control. (D) Quantified phosphorylated (p‐) to total AKT^(T308)^, rpS6^(S240/244)^ and 4EBP1^(T37/44)^ protein expression. (E) Experiment overview: C2C12 myoblasts are plated on Type I collagen‐coated plates in growth medium (10% FBS) and switched to DM (2% HS) after 48 h, which corresponds to Day 0 of differentiation. At Day 5 of differentiation, C2C12 myotubes were switched to high‐serum growth medium (GM, 5% FBS) or LLC medium for 48 h. C2C12 myotubes were rinsed with PBS and switched to minimal essential medium (MEM) for 1 h on Day 7 of differentiation and then treated with vehicle or 50‐mM leucine in MEM for 60 min. Representative Western blotting images with Ponceau S stain as a protein loading control. (F) Quantified phosphorylated (p‐) to total AKT^(T308)^, rpS6^(S240/244)^ and 4EBP1^(T37/44)^ protein expression. Data are presented as mean ± SEM. Data are analysed using unpaired *t*‐test or two‐way ANOVA with Tukey's post hoc. Statistical significance was set to *p* < 0.05. *N* = 6 per group from two independent experiments. #Main effect (ME) of LLC. ‘a’, ‘b’ and ‘c’ mean significant difference between groups.

We used an in vitro myotube model to understand cancer‐conditioned media and anabolic signalling. We examined C2C12 myotubes at Day 5 of differentiation, and differentiation media (DM) was changed to 50% LLC conditioned media (CM) or 50% growth media (GM) as a control for high serum cancer cell culture conditions for 48 h (Figure 3A). In agreement with prior studies, LLC CM reduced myotube diameter, decreased myosin heavy chain and 4EBP1 phosphorylation and increased STAT3^(Y705)^ phosphorylation and atrogin‐1 compared with GM myotubes (Figure 3B). LLC CM did not change gp130 protein expression (Figure 3B). Insulin administration acutely increased AKT^(T308)^ and rpS6^(S240/244)^ phosphorylation in GM and LLC CM myotubes (Figure 3C,D). Insulin AKT induction was lowered in the LLC CM myotubes (Figure 3D). As expected, leucine did not alter AKT^(T308)^ or rpS6^(S240/244)^ phosphorylation but increased 4EBP1^(T37/44)^ phosphorylation in GM myotubes (Figure 3E,F).

### 3.4 Effect of Muscle‐Specific gp130 Loss on Skeletal Muscle Anabolic Signalling

As the feeding‐induced skeletal muscle anabolic response was maintained in cachectic LLC mice, we investigated the impact of the muscle‐specific deletion of gp130 (Gp130mKO) on the LLC fasting suppression of skeletal muscle anabolic signalling. Gp130 floxed (gp130WT) and gp130 floxed tamoxifen‐inducible HSA‐MCM (gp130mKO) male mice received daily injections of tamoxifen for 5 days to induce recombination (Figure S2A). Mice were subsequently given a 2‐week washout period, and then, mice received a subcutaneous injection of LLC cells or PBS. All mice were euthanized in the fasted condition (Figure 5A).

Gp130mKO did not alter tumour burden or prevent muscle atrophy in LLC mice (Table 2). Gp130mKO mice weighed less compared with WT mice (Table 2). Independent of genotype, the LLC mice had greater body weight loss and reduced fat mass but only demonstrated a trend for decreased muscle mass compared with PBS (Table 2). LLC mice had splenomegaly and elevated plasma IL‐6 levels compared with PBS. Gp130mKO did not alter fasting blood glucose and plasma insulin levels; however, LLC mice, independent of genotype, had increased blood glucose levels compared with PBS mice (Table 2).

**TABLE 2:** Effect of muscle‐specific gp130 loss on animal characteristics in LLC tumour–bearing mice under fasting condition.

	PBS		LLC		p		
	WT	mKO	WT	mKO			
*N*	7	7	9	9	mKO × LLC	ME mKO	ME LLC
BW D0 (g)	26.6 (0.5)	25.0 (0.9)	26.9 (0.5)	25.2 (0.5)	0.901	***0.022***	0.691
BW D10 (g)	27.8 (0.6)	25.6 (0.8)	27.6 (0.7)	26.2 (0.6)	0.519	***0.016***	0.736
Tumour‐free final BW (g)	28.1 (0.6)	27.0 (0.9)	26.2 (0.6)	24.5 (0.5)	0.648	0.053	***0.003***
BW change from D0 (%)	5.8 (1.8)	8.1 (1.4)	−2.5 (2.3)	−2.6 (1.6)	0.526	0.557	***< 0.001***
BW change from D10 (%)	1.0 (0.7)	5.8 (1.3)	−4.8 (3.0)	−6.3 (1.6)	0.087	0.368	***< 0.001***
Tumour weight (g)	—	—	3.3 (0.8)	3.8 (0.7)	—	—	0.669
TGR (cm^3^/5 days)	—	—	0.4 (0.1)	0.3 (0.1)	—	—	0.356
Gastrocnemius (mg)	127.7 (4.3)	121.3 (1.8)	120.7 (4.6)	112.5 (3.8)	0.816	0.052	0.071
HLM (mg)	201.0 (6.9)	194.9 (4.4)	194.7 (9.1)	178.7 (6.0)	0.474	0.116	0.108
eWAT (mg)	310.3 (33.5)	479.0 (70.2)^a^	240.3 (31.7)^b^	202.7 (32.1)^c^	***0.038***	0.201	***< 0.001***
Blood glucose (mg/dl)	96.4 (7.6)	103.6 (7.3)	126.7 (13.6)	125.9 (9.2)	0.686	0.745	***0.012***
Plasma insulin (μg/L)	0.4 (0.1)	0.4 (0.2)	0.3 (0.1)	0.3 (0.1)	0.795	0.573	0.306
IL‐6 (pg/mL)	4.6 (0.7)	5.2 (1.3)	17.5 (2.1)	12.4 (3.5)	0.314	0.412	***0.004***
Spleen (g)	78 (3.6)	72.7 (3.7)	193.7 (26.6)	219 (23.9)	0.416	0.588	***< 0.001***
Tibia (mm)	17.3 (0.1)	17.2 (0.1)	17.1 (0.1)	17.2 (0.1)	0.316	0.224	0.685

*Note:* Data are presented as mean ± (SEM). Data are analysed using two‐way ANOVA with Tukey's multiple comparisons test. Statistical significance is set to *p* < 0.05. Bold and italicized text denotes a significant *p*‐value.

Abbreviations: μg/L: microgram per litre; BW: body weight; D0: day begin tumour inoculation; D10: Day 10 post tumour inoculation; eWAT: epidydimal white adipose tissue; g: grams; HLM: hindlimb muscle mass includes gastrocnemius, soleus, plantaris, tibialis anterior and extensor digitorum longus; LLC: Lewis Lung Carcinoma; mg/dL: milligrams per decilitre; mg: milligrams; mm: millimetres; PBS: phosphate‐buffered saline; pg/mL: picograms per millilitre; TGR: tumour growth rate.

^a^ Different from PBS‐WT.

^b^ Different from PBS‐mKO.

^c^ Different from LLC‐PBS.

**FIGURE 4:**
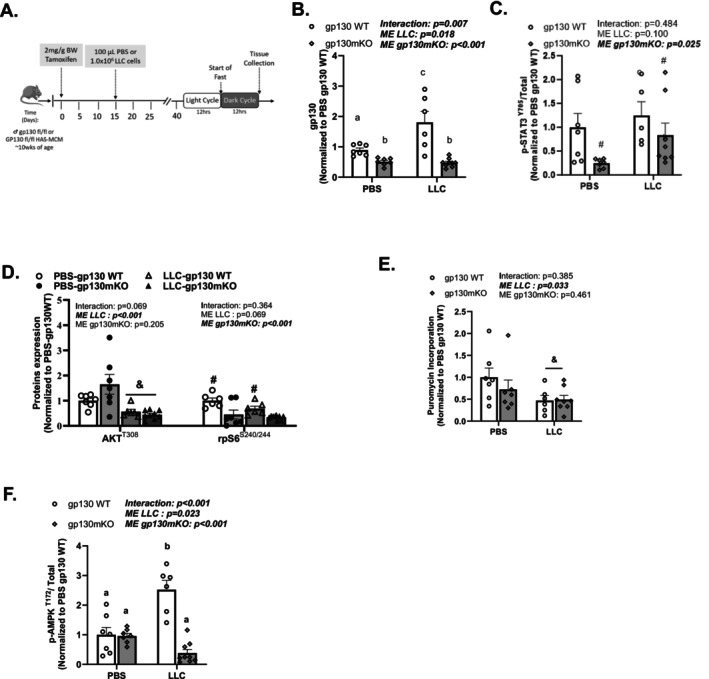
Muscle‐specific gp130 loss is sufficient to reduce elevated AMPK, without improving the suppression of mTORC1 signalling and protein synthesis in LLC male mice. (A) Study design: gp130 floxed and gp130 floxed tamoxifen inducible HSA Mer‐Cre‐Mer (MCM) mice were given an injection daily of tamoxifen. Following the 5th day of tamoxifen, mice were subsequently given a 2‐week washout period prior to PBS or LLC cell injection. Mice were sacrificed in fast condition. (B) gp130 protein expression. Quantified phosphorylated (p‐) to total (C) STAT3^(Y705)^ and (D) AKT^(T308)^, rpS6^(S240/244)^ protein expression. (E) Puromycin incorporation. (F) AMPK^(T172)^ protein expression. Data are presented as mean ± SEM. Data are analysed using two‐way ANOVA with Tukey's post hoc. Statistical significance was set to *p* < 0.05. *N* = 7–9 per group. #Main effect (ME) of gp130mKO. &Main effect (ME) of LLC. ‘a’, ‘b’ and ‘c’ mean significant difference between groups.

Muscle gp130 protein expression was reduced by approximately 40% in gp130mko, and downstream STAT3^(Y705)^ phosphorylation was reduced compared with their floxed littermates (Figure 4B,C). This level of knockdown was expected as muscle tissue contains a variety of cell types expressing gp130. Gp130mKO did not restore LLC‐fasting suppression of either AKT^(T308)^ or rpS6^(S240/244)^ phosphorylation and decreased protein synthesis (Figure 4D,E). Interestingly, gp130mKO restored LLC‐induced AMPK^(T172)^ phosphorylation (Figure 4F). In summary, muscle‐specific gp130 loss reduced the aberrant LLC‐fasting AMPK induction but did not rescue muscle anabolic signalling in tumour‐bearing mice.

### 3.5 Effect of Muscle‐Specific AMPK Loss on Skeletal Muscle Anabolic Signalling

**FIGURE 5:**
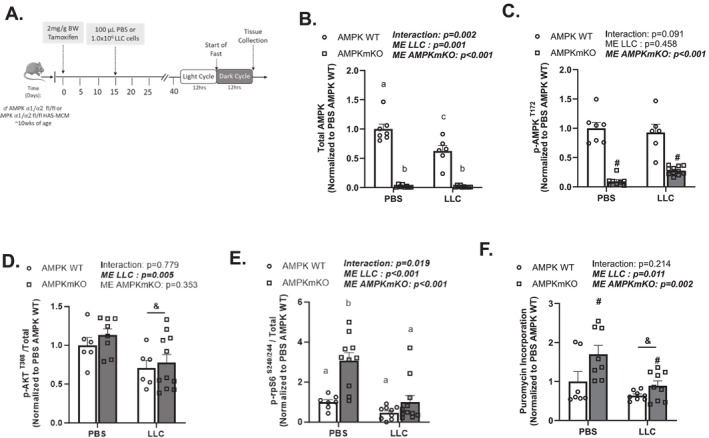
Muscle‐specific AMPK loss improves the fasting suppression of mTORC1 signalling and protein synthesis in LLC mice initiating cachexia. (A) Study design: AMPKa^1^a^2^ floxed and AMPKa^1^a^2^ floxed tamoxifen‐inducible HAS‐MCM mice were given an injection daily of tamoxifen. Following the 5th day of tamoxifen, mice were subsequently given a 2‐week washout period prior to PBS or LLC cell injection. Mice were sacrificed in the fast condition. (B and C) Total and phosphorylated AMPK^(T172)^ protein expression. Quantified phosphorylated (p‐) to total (D) AKT^(T308)^ and (E) rpS6^(S240/244)^ protein expression. (F) Puromycin incorporation. Data are presented as mean ± SEM Data are analysed using two‐way ANOVA with Tukey's post hoc. Statistical significance was set to *p* < 0.05. *N* = 7–11 per group. #Main effect (ME) of AMPKmKO. &Main effect (ME) of LLC. ‘a’, ‘b’ and ‘c’ mean significant differences between groups.

Fasting can induce muscle AMPK activation while inhibiting mTORC1 signalling and protein synthesis. Therefore, we investigated muscle‐specific AMPK loss (AMPKmKO) in LLC mice. AMPKα^1^/α^2^ floxed (AMPKWT) and AMPKα^1^/α^2^ floxed tamoxifen‐inducible HSA‐MCM (AMPKmKO) mice received daily injections of tamoxifen for five consecutive days (Figure S1B). After a 2‐week washout period, mice were randomized into the LLC or PBS. All mice were euthanized in the fasted condition (Figure 5A).

Interestingly, we report that AMPKmKO was sufficient to slow tumour growth and tumour volume at the end of the study (Table 3). Additionally, AMPKmKO mice had reduced muscle mass compared with WT controls (Table 3). Body weights were similar between AMPKWT and AMPKmKO mice at the start and on Day 10 of LLC inoculation. Despite the reduction in LLC tumour size in AMPKmKO mice, neither muscle wasting nor body weight loss was affected (Table 3). There were no differences in fat mass. LLC mice, independent of genotype, had splenomegaly and elevated plasma IL‐6 levels compared with PBS mice.

**TABLE 3:** Effect of muscle‐specific AMPK loss on animal characteristics in LLC tumour–bearing mice under fasting condition.

	PBS		LLC		*p*		
	WT	mKO	WT	mKO			
*N*	7	10	9	11	mKO × LLC	ME mKO	ME LLC
BW D0 (g)	25.7 (0.8)	25.4 (0.5)	26.0 (0.5)	25.5 (0.6)	0.889	0.459	0.744
BW D10 (g)	27.3 (0.6)	26.5 (0.6)	26.8 (0.6)	25.9 (0.6)	0.963	0.243	0.429
Tumour‐free final BW g)	29.0 (0.7)	27.4 (0.4)	25.5 (0.6)	25.0 (0.5)	0.319	0.080	***< 0.001***
BW change from D0 (%)	14 (3.7)	8.2 (1.5)	−1.8 (2.6)	−1.5 (1.7)	0.198	0.242	***< 0.001***
BW change from D10 (%)	7.4 (2.8)	3.5 (1.4)	−4.6 (2.0)	−3.3 (1.6)	0.175	0.493	***< 0.001***
Tumour weight (g)	—	—	4.6 (0.6)	2.8 (0.5)	—	—	***0.037***
TGR (cm^3^/5 days)	—	—	0.4 (0.1)	0.2 (0.0)	—	—	***0.010***
Gastrocnemius (mg)	127.7 (3.2)	113.7 (2.6)	108 (4.0)	103.6 (4.1)	0.273	***0.027***	***< 0.001***
HLM (mg)	211.9 (5.0)	193.7 (4.2)	179.8 (5.6)	175.2 (5.6)	0.257	0.053	***< 0.001***
eWAT (mg)	343.3 (34.7)	387.3 (61.2)	250.7 (38.7)	293.6 (55.3)	0.991	0.417	0.087
Blood glucose (mg/dl)	105.1 (9.3)	95.4 (6.1)	95.0 (9.9)	85 (5.7)	0.987	0.204	0.186
Plasma insulin (μg/L)	0.2 (0.0)	0.2 (0.0)	0.2 (0.0)	0.3 (0.1)	0.839	0.590	0.159
IL‐6 (pg/ml)	5.8 (1.9)	5.8 (1.2)	17.2 (4.1)	12.7 (2.1)	0.365	0.357	***< 0.001***
Spleen (g)	77.6 (6.8)	69.4 (3.5)	244.0 (27.3)	167.6 (18.3)	0.064	***0.023***	***< 0.001***
Tibia (mm)	17.3 (0.1)	17.2 (0.2)	16.7 (0.2)	17.1 (0.1)	0.087	0.262	***0.046***

*Note:* Data are presented as mean ± (SEM). Data are analysed using two‐way ANOVA with Tukey's multiple comparisons test. Statistical significance is set to *p* < 0.05. Bold and italicized text denotes a significant *p*‐value.

Abbreviations: μg/L: microgram per litre; BW: body weight; D0: day begin tumour inoculation; D10: Day 10 post tumour inoculation; eWAT: epididymal white adipose tissue; g: grams; HLM: hindlimb muscle mass includes gastrocnemius, soleus, plantaris, tibialis anterior and extensor digitorum longus; LLC: Lewis Lung Carcinoma; mg/dL: milligrams per decilitre; mg: milligrams; mm: millimetres; PBS: phosphate‐buffered saline; pg/mL: picograms per millilitre; TGR: tumour growth rate.

Muscle phospho‐AMPK and total AMPK were significantly reduced in AMPKmKO mice (Figure 5B–C). As expected, AKT^(T308)^ phosphorylation was not affected by AMPKmKO in PBS or LLC mice; however, LLC did suppress AKT^(T308)^ regardless of genotype (Figure 5D). In PBS mice, rpS6^(S240/244)^ phosphorylation was increased by AMPKmKO and also suppressed by LLC (Figure 5E). There was a main effect for AMPKmKO to increase puromycin incorporation, regardless of PBS or LLC treatments (Figure 5F). There was also a main effect for LLC to reduce puromycin incorporation regardless of genotype. In conclusion, skeletal muscle AMPKα^1^/α^2^ loss in LLC male mice did not restore the fasting suppression of mTORC1 but did induce protein synthesis.

## 4 Discussion

Cancer cachexia is a debilitating whole‐body wasting condition, and disrupted skeletal muscle metabolic homeostasis has a critical role in driving this catabolism. Studies examining the regulation of skeletal muscle protein turnover during cancer cachexia have often either not controlled for feeding, performed a brief fasting period or not defined the extent of the fasting before examining skeletal muscle signalling. This study design issue has likely led to inconsistencies and equivocal findings when examining anabolic signalling regulation in preclinical cancer models. Because of the tight linkage with diurnal oscillations in muscle AMPK and mTORC1 signalling pathways connected to feeding, fasting and cage activity, it is crucial to understand cytokine‐initiated regulation of these pathways in the context of controlled feeding and fasting conditions. Therefore, we investigated the feeding and fasting regulation of skeletal muscle protein synthesis signalling in tumour‐bearing male mice. We report the novel finding that the cancer environment does not impede skeletal muscle's response to feeding. At least in the established LLC mouse model, anabolic resistance to a food stimulus does not appear to be a driver of cachexia; however, further research is required to determine the anabolic response in tumour‐bearing female mice. We also report the intriguing finding that LLC male mice have a disrupted metabolic response to a prolonged fast when compared with healthy mice. This finding aligns with and extends prior studies in multiple preclinical cancer models, finding that defined periods of fasting activate muscle AMPK and suppress mTORC1 signalling. In agreement with our previous work, LLC tumour–bearing mice exhibit an accelerated fasting suppression of mTORC1 and activation of AMPK in skeletal muscle [28]. As the IL‐6 family of cytokines has been extensively examined in several preclinical cancer models, we report the intriguing finding that muscle‐specific loss of gp130 could prevent the exacerbated fasting induction of AMPK phosphorylation. Therefore, the IL‐6 family of cytokines appears to have a role in the upstream regulation that disrupts fasting muscle AMPK activity during cachexia. Interestingly, gp130 loss in skeletal muscle did not restore cancer suppression of fasting protein synthesis. However, we provide evidence for altered AMPK activity in the cancer disruption of protein turnover. In tumour‐bearing mice, muscle‐specific AMPK loss partially restored fasting‐induced suppression of anabolic signalling. Beyond investigating basic AMPK regulation of intracellular signalling, it is important to understand that chronic AMPK suppression in muscle has negative consequences that are often severe. Muscle‐specific over‐expression of a dominant negative AMPK can promote cachexia and metabolic dysfunction in LLC tumour–bearing mice [29].

We report that male LLC tumour–bearing mice can still induce muscle protein synthesis in response to feeding. Similarly, in Apc^Min/+^ mice, a genetic colon cancer cachexia model, suppressed anabolic signalling is associated with cachexia [13, 30] and acute glucose administration can induce skeletal muscle anabolic signalling [12]. Interestingly, Apc^Min/+^ mice also have disrupted diurnal feeding, fasting and cage activity behaviours that alter muscle mTORC1 signalling [14]. We extend these findings using the LLC tumour model by reporting that glucose, but not insulin, was increased in the LLC compared with PBS despite similar food consumption. Similarly, feeding in LLC tumour–bearing mice increased mTORC1 signalling but not AKT. Interestingly, a previous study reported increased muscle AKT^(S473)^ following glucose administration in Apc^Min/+^ mice, which is in contrast to the inability of LLC‐fed mice to increase AKT^(T308)^ in our current study [12]. Because of mTORC1 being an established critical node for integrating muscle anabolic and catabolic signalling, further investigation is needed to determine additional mTORC1‐regulated processes (autophagy targets, ribosome biogenesis targets) controlling muscle responsiveness during cachexia. This knowledge should provide critical context for interpreting the effects of fasting and refeeding [28].

Although it is well established in healthy muscle that feeding‐induced anabolic signalling can occur by insulin‐induced AKT activation, an interesting future line of inquiry would be to assess if the increased amino acid availability could regulate mTORC1 and protein synthesis independent of insulin. Additionally, there is a need to establish if both insulin and amino acid availability are required to synergistically induce muscle protein synthesis in tumour‐bearing mice. We report that mTORC1 signalling and protein synthesis were suppressed in LLC tumour–bearing mice, independent of the fast or fed condition. Given that our data and others have shown that cachectic skeletal muscle response to various anabolic stimuli is intact, the data suggest a critical role for an accelerated fasting response in cancer‐induced muscle wasting that warrants further investigation. The goal of our study was to examine a short‐term, defined period of feeding using an established animal model for investigating the muscle anabolic and catabolic response [9]. An important future line of inquiry would be to expand the feeding stimulus beyond standard rodent chow to involve defined diets, single amino acids, and supplements for both short‐term and long‐term (e.g., 24 h) regulation of muscle anabolism. Furthermore, using techniques that can investigate the effect of cancer on the ad libitum food anabolic response over a longer time, including the entire dark cycle, would provide critical context for interpreting the anabolic and catabolic effects of fasting and feeding. As the current study was delimited to males, the investigation of the female response is required to understand the clinical and translational ramifications of the anabolic flexibility reported in our study.

Muscle AMPK signalling is an established regulator of skeletal muscle metabolism and muscle protein turnover in response to metabolic challenges [8]. Moreover, there is a growing interest in the potential role of AMPK activation as a protector of tissue during wasting and potentially enhancing anabolic processes [8]. Elevated AMPK levels have been reported in skeletal muscle of patients with non‐small cell lung cancer (NSCLC), and the increase was associated with cachexia [29]. Previously, we reported that fasting skeletal muscle AMPK is chronically elevated in cachectic Apc^Min/+^ and LLC mice [12, 24]. In addition, our recent data defined the muscle's catabolic response to fasting and found that AMPK was suppressed compared with ad libitum‐fed mice [27]. The current study extended our prior work by incorporating the fed response and identified that 1 h of feeding can reduce AMPK activation in tumour‐bearing mice before cachexia. There are established adverse effects of AMPK loss in cardiac and skeletal muscle studies, and AMPK activators have been shown to benefit muscle metabolism in many conditions [31, 32]. Importantly, under inflammatory conditions, the impact of AMPK loss is less studied [31, 32], which is often due to the investigations being designed to examine the induction of AMPK activity by pharmacological or exercise interventions. AMPK activation has established beneficial effects in several chronic diseases [8]. However, it is critically important to account for the specific disease condition, such as cancer cachexia, which can exhibit chronically elevated AMPK signalling [12].

The available evidence clearly demonstrates that there is no benefit and possibly severe negative consequences related to AMPK inhibition in cachectic skeletal muscle. AMPK activity loss in LLC tumour–bearing mice using over‐expression of a dominant‐negative AMPKα2 has been reported to accelerate the cachexia phenotype [29]. Furthermore, Raun et al. reported that AMPK loss aggravated LLC‐induced metabolic dysfunction. The effect of whole‐body AMPK activation on cachexia has been investigated with AMPK activators, AICAR and Metformin. Administered before cachexia, AICAR treatment reduced muscle mass loss in tumour‐bearing mice [33, 34] and lowered E3 ligase gene expression and autophagy [34]. Metformin has also been shown to prevent cachexia [33, 35]. We are incredibly interested in the cancer‐induced disruptions upstream of AMPK that cause aberrant metabolic regulation in fasted condition. Our investigation points to the potential for inflammatory signalling to disrupt muscle metabolic signalling, including AMPK responses to fasting. There is also a need to investigate the role of aberrant AMPK signalling in response to other anabolic stimuli, such as muscle contraction and resistance exercise during cancer cachexia. The cause of the chronic induction of muscle AMPK in cachexia is not known, but it is interesting to propose that it may be due to a heightened fasting metabolic response or other metabolic dysfunctions caused by cancer. As mitochondrial loss and dysfunction are established drivers of LLC‐cachexia [36], it is intriguing to speculate that aberrant AMPK regulation may be driven by disruptions to muscle mitochondria. These disruptions could include aberrant ROS and mitochondria quality control regulation, which are worthy of further investigation.

Prior work from our lab has shown that muscle‐specific deletion of gp130 can prevent muscle wasting in tumour‐bearing mice [24]. In the current study, muscle mass was not preserved by knocking out muscle gp130, but muscle mass loss was extremely mild. There are several considerations in examining the outcomes related to muscle‐specific gp130 loss. First, our prior study employed a different muscle‐specific promoter, myosin light chain, which was not inducible and did not involve tamoxifen administration. In contrast, the current study used the extensively employed HSA‐inducible model. Our prior study produced a more robust weight loss overall compared with our current results. Notably, we used younger mice in the prior study, initiating the study at 8 weeks of age, rather than 12 weeks of age as in the current study. This 4‐week age difference is at a critical time of rapid growth in mice and could impact the interaction of growth suppression by the tumour. Finally, circulating IL‐6 was lower in our current study compared with our prior study. The inflammatory environment contributes to muscle wasting, and muscle gp130's regulatory effects may be impacted by the type and concentration of the IL‐6 family of cytokines in LLC mice. Intracellular signalling initiated by gp130 can regulate growth, differentiation and apoptosis [21]. Importantly, gp130 loss prevented LLC‐induced skeletal muscle p‐AMPK. Prior work showed that LLC‐induced AMPK did not require gp130 in more cachectic mice than in the current study [24]. It is interesting to speculate that during cachexia, additional stimuli upstream of AMPK, such as energy stress, mitochondria dysfunction or ROS, may be involved in aberrant muscle AMPK regulation independent of cytokine signalling. We and others have shown that gp130 [37] and AMPK [38] can regulate mitophagy. It is reasonable to suggest that during cachexia, mitophagy is disrupted, and by inhibiting gp130, we can prevent this perturbation. As the current study was conducted over 25–30 days, research is needed to determine whether gp130 alters muscle metabolic regulation during the time course of cachexia and whether muscle phenotype has a role in the response. Furthermore, we have found that female mice have a differential response to IL‐6 and the development of cachexia involving intact ovary function [39]. The role of gp130 signalling in female cancer cachexia needs further study to disentangle the direct and indirect effects of muscle‐specific gp130 in cachexia.

In summary, we report the novel finding that skeletal muscle from LLC tumour–bearing males can respond to an anabolic feeding stimulus, and anabolic resistance to feeding does not appear to regulate cachexia in these mice. Feeding increased AKT/mTORC1 signalling and protein synthesis in LLC tumour–bearing male mice. As the current study did not contain a time course of cachexia development, further research is warranted to determine if the muscle anabolic response to feeding could occur during the stage of pre‐cachexia. We also report that LLC tumour–bearing mice have an exacerbated catabolic response to a prolonged fast when compared with healthy mice, which involves suppressed muscle AKT/mTORC1 signalling and protein synthesis. Interestingly, muscle gp130 loss, which attenuates the IL‐6 family of cytokine signalling in muscle, was sufficient to rescue the aberrant fasting regulation of muscle AMPK. Further research is warranted to establish the IL‐6 cytokine family control of the aberrant upstream and downstream regulation of muscle AMPK activity that is disrupted with cancer and leads to aberrant protein turnover regulation with cachexia.

## Ethics Statement

The authors comply with the ethical guidelines for authorship and publishing in the Journal of Cachexia, Sarcopenia and Muscle [40].

## Conflicts of Interest

The authors declare no conflicts of interest.

## Supporting information


**Table S1:** Effects of feeding on stomach weight.
**Table S2:** Correlation in fasting LLC tumour–bearing mice.
**Table S3:** Antibodies used for immunoblotting analysis.
**Figure S1:** Cachexia in LLC tumor‐bearing mice.
**Figure S2:** C2C12 myotubes treated with LLC conditioned media alters the mTORC1 response to insulin and leucine.
**Figure S3:** Cachexia in gp130 LLC tumor‐bearing mice.
**Figure S4:** Cachexia in AMPK LLC tumor bearing mice.
