# Supplementary material for: The Effect of Cachexia on the Feeding Regulation of Skeletal Muscle Protein Synthesis in Tumour‐Bearing Mice
Source: J Cachexia Sarcopenia Muscle. 2025 Sep 10;16(5):e70064. doi: 10.1002/jcsm.70064 (PMC12423108; doi:10.1002/jcsm.70064)
Supplement: Supplementary file 1 — Table S1: Effects of feeding on stomach weight. Table S2: Correlation in fasting LLC tumour–bearing mice. Table S3: Antibodies used for immunoblotting analysis. Figure S1: Cachexia in LLC tumor‐bearing mice. Figure S2: C2C12 myotubes treated with LLC conditioned media alters the mTORC1 response to insulin and leucine. Figure S3: Cachexia in gp130 LLC tumor‐bearing mice. Figure S4: Cachexia in AMPK LLC tumor bearing mice. [file JCSM-16-e70064-s001.docx]

**The Effect of Cachexia on the Feeding Regulation of Skeletal Muscle Protein Synthesis in Tumor-Bearing Mice**

# Methods

**Cell Culture**

All cells were purchased through ATCC (Manassas, VA) and used after the 5^th^ passage and within the 10th passage. LLC (CRL-1462) and murine C2C12 myoblasts (CRL-1772) cells were cultured at 37C, 5% CO_2_ in growth media (GM): Dulbecco’s Modified Eagle Medium (DMEM; Gibco, no. 11995-065) supplemented with 10% fetal bovine serum (FBS; Gibco, no. A52094-01), 50 U/mL penicillin, and 50 lg/mL streptomycin (Gibco, no. 15140-122) as previously described [1, 2].

**C2C12 Myotube Differentiation**

C2C12 myoblasts were seeded on type I collagen-coated polystyrene plastic at a density of 9.0 x10^4^ cells per well (six-well plate) in GM. To induce myoblast differentiation, cells were rinsed with phosphate-buffered saline (PBS) and switched to differentiation media (DM): DMEM supplemented with 2% heat-inactivated horse serum, 50 U/ml of penicillin, and 50 mg/ml of streptomycin to form myotubes. Media was replenished every 48 h, and experiments were performed starting at day 5 of differentiation when multinucleated contractile myotubes are present.

**LLC Conditioned Media Collection**

Conditioned media (CM) consists of secreted factors from LLC tumor cells and has been described previously [1]. Briefly, 2x10^6^ cells were seeded in 150-mm tissue culture-treated plates in GM. Tumor cell CM was collected at 90% confluence 48-hr post cell seeding and spun down at 3,000 rpm for 5 min to remove cell debris. Cells on the plate were pelleted and counted via trypan blue exclusion test to ensure an equivalent number of cells on the plate, with a final density averaging 7.0–9.0x10^6^ cells per culture dish. CM was stored for one-time use in aliquots at -20 °C, used within 2 months, and then thawed in a warm water bath at the time of the experiment. GM with no cells was used as media control.

**Treatment of myotubes**

On day 5 of differentiation, C2C12 myotubes were treated with GM control or LLC CM diluted with serum-free DMEM for a final serum concentration of 5% FBS. GM control and LLC CM were replenished after 24-hr. In a separate experiment, differentiated myotubes on day 5 were treated with 50% GM or 50% LLC CM for 48-hr. After 48-hr, the media was removed, and cells were placed in minimal essential media to quiesce the myotubes. After 60 minutes, 20nM of insulin or 5mM leucine was dosed on the cells for 60 minutes, and then cells were collected for Western blot analysis.

**Myotube Diameter**

Myotube diameter was quantified as previously described [3] with the following modifications. C2C12 myotube diameter was quantified using ImageJ software (National Institutes of Health, Bethesda, MD, United States). Digital images were captured at X20 objective brightfield. Five non-overlapping images were captured within each well, and three images were randomly chosen for the analysis. The analysis used unmodified tiff images accessed in NIH ImageJ software. A blinded investigator randomly took diameter measurements of six myotubes per image based on preset inclusion/exclusion criteria: elongated structure with distinct membrane outlines, little to no cellular debris, and no branching points. The average diameter per myotube was calculated as the mean of eight measurements taken along the myotube length.

**Generation of muscle-specific deletion mice**

Tamoxifen-inducible Mer Cre Mer driven by human skeletal actin promoter (HSA-MCM) mice were purchased from Jackson Laboratories (Bar Harbor, ME, USA). Mice on a C57BL/6 background that contained individually floxed alleles for AMPKα1and AMPKα2 were provided by Dr. Hoh-Jin Koh at the University of South Carolina. Mice on a C57BL/6 background that contained floxed alleles for gp130 were provided by Dr. Colin Stewart’s laboratory [Laboratory of Cancer and Developmental Biology, National Cancer Institute, U.S. National Institutes of Health (NIH), Frederick, MD, USA] in collaboration with Dr. Lothar Hennighausen [Laboratory of Genetics and Physiology, National Institute of Diabetes and Digestive and Kidney Diseases, NIH, Bethesda,MD, USA] [4]. Gp130 or AMPKa1a2 floxed mice crossed with HSA-MCM mice and male tamoxifen-inducible gp130mKO and AMPKmKO were produced. To control for tamoxifen, all floxed and mKO mice received tamoxifen injections (I.P. 2mg) once daily for 5 consecutive days. Then, they underwent a 2-week washout period before PBS or LLC injections.

**Lewis Lung Carcinoma (LLC) Cell Inoculation**

Between 11-12 wks. of age (B6) and 13-14 wks. of age (mKO) mice were injected with either phosphate buffered saline (PBS) or 1 x 10^6^ LLC cells subcutaneously in the right flank under anesthesia. A mouse reached the studies endpoint if the following criteria were met (1) mouse reached 30 days post tumor inoculation, (2) >20% body weight loss from day 10 after day 25, or (3) had a tumor >3cm in width or length after day 25, or (4) the tumor was close to breaking through the skin (ulcerated) after day 25. Once an endpoint was achieved, the mouse was prepared for tissue collection and euthanized within 24 hrs. At day 21, 1 mouse had an ulcerated tumor and was not included in the analysis and 1 mouse died unexpectedly at day 24. To be included in the study, mice needed to achieve at least 25 days post-tumor inoculation. Tumor volume and body weight were measured every 5 days to calculate tumor growth. Tumor volume was calculated using the following equation: ½ (width^2^xlength) [5].

**Western Blot**

We ran three different gels for all the samples. The strategy for western blot analysis is as follows: the signals from phosphorylation antibodies were expressed relative to total protein on the same gel and quantified as phosphorylation to total ratio and then PBS fast in PBS and Fast gel; Normalized by total protein first and then LLC fast in LLC gel, then further normalized to PBS Fast. This allowed for comparison between gels and the 2-way ANOVA analysis.

1. Halle JL, Counts-Franch BR, Prince RM, Carson JA. The Effect of Mechanical Stretch on Myotube Growth Suppression by Colon-26 Tumor-Derived Factors. Front Cell Dev Biol. 2021;9:690452. doi:10.3389/fcell.2021.690452

2. Zhang Q, Halle JL, Counts BR, Pi M, Carson JA. mTORC1 and BMP-Smad1/5 regulation of serum-stimulated myotube hypertrophy: a role for autophagy. Am J Physiol Cell Physiol. 2024;327:C124-C39. doi:10.1152/ajpcell.00237.2024

3. Gao S, Carson JA. Lewis lung carcinoma regulation of mechanical stretch-induced protein synthesis in cultured myotubes. Am J Physiol Cell Physiol. 2016;310:C66-79. doi:10.1152/ajpcell.00052.2015

4. Zhao L, Hart S, Cheng J, Melenhorst JJ, Bierie B, Ernst M, et al. Mammary gland remodeling depends on gp130 signaling through Stat3 and MAPK. J Biol Chem. 2004;279:44093-100. doi:10.1074/jbc.M313131200

5. Jensen MM, Jorgensen JT, Binderup T, Kjaer A. Tumor volume in subcutaneous mouse xenografts measured by microCT is more accurate and reproducible than determined by 18F-FDG-microPET or external caliper. BMC Med Imaging. 2008;8:16. doi:10.1186/1471-2342-8-16

**Supplementary Table 1. Effects of feeding on stomach weight**

|  | **PBS** | | **LLC** | |
| --- | --- | --- | --- | --- |
|  | **Fast** | **Fed** | **Fast** | **Fed** |
| **N** | 7 | 9 | 10 | 14 |
| **Stomach Mass (mg)** | 375 (41) ^a^ | 855 (112) ^b^ | 390 (32) ^a^ | 573 (41) ^a^ |
| **Empty Stomach (mg)** | 206 (12) | 158 (8) | 180 (8) | 135 (6) |
| **Stomach Content (mg)** | 169 (33) ^a^ | 697 (114) ^b^ | 209 (26) ^a^ | 434 (43) ^c^ |

Data is presented as Mean (SEM). Abbreviations: PBS: phosphate buffered saline; LLC: Lewis Lung Carcinoma; mg: milligrams. Two-way ANOVAs were used to compare PBS and LLC in the Fast and Fed condition. # Main Effect of Fed. $ Main effect of LLC. If an interaction was present, different letters were used to differentiate differences between groups. Statistically significant *p*<0.05.

**Supplementary Table 2: Correlation in fasting LLC tumor-bearing mice**

|  | **p/t_Akt** | **p/t_rpS6** | **Puromycin** | **p/t_Ampk** |
| --- | --- | --- | --- | --- |
| **BW change from D10 (%)** | 0.329 | 0.680 | 0.560 | 0.233 |
| **HLM (mg)** | -0.501 | -0.356 | 0.288 | -0.125 |
| **eWAT (g)** | -0.041 | -0.449 | 0.300 | 0.200 |
| **Spleen (g)** | -0.308 | -0.379 | -0.521 | -0.054 |
| **Tibia (mm)** | -0.471 | -0.138 | 0.380 | -0.034 |
| **Tumor mass (g)** | -0.051 | -0.248 | ***-0.780**** | -0.185 |
| **TGR (cm^3^/5 days)** | 0.091 | ***-0.765**** | -0.610 | -0.267 |
| **Glucose(mg/dl)** | 0.354 | -0.108 | 0.204 | -0.289 |
| **Insulin (ug/L)** | ***0.888**** | -0.003 | -0.332 | -0.455 |
| **IL-6 (pg/ml)** | 0.482 | 0.114 | -0.511 | 0.046 |

Abbreviations: LLC: Lewis Lung Carcinoma; BW: body weight; D10: day 10 post tumor inoculation; HLM: total hindlimb muscle mass; eWAT: epidydimal white adipose tissue; TGR: Tumor growth rate; g: grams; mg: milligrams; mm: millimeters; mg/dl: milligrams per deciliter; ug/L: microgram per liter; pg/ml: picograms per milliliter. Spearman correlation coefficient was used to determine associations. * *p*<0.05.

**Supplementary Table 3. Antibodies used for immunoblotting analysis.**

| ***Antibody*** | ***Cat#*** | ***dilution*** | **Supplier** |
| --- | --- | --- | --- |
| **Phosphorylated rpS6 (S240/244)** | #5364 | 1:1000 | Cell Signaling Technology |
| **rpS6** | #2217 | 1:2000 | Cell Signaling Technology |
| **Phosphorylated Akt (T308)** | #13038S | 1:500 | Cell Signaling Technology |
| **Akt** | #9272S | 1:2000 | Cell Signaling Technology |
| **Phosphorylated AMPK (T172)** | #2535S | 1:1000 | Cell Signaling Technology |
| **AMPK** | #2630S | 1:2000 | Cell Signaling Technology |
| **Phosphorylated STAT3 (Y705)** | #9145S | 1:1000 | Cell Signaling Technology |
| **STAT3** | #4904S | 1:2000 | Cell Signaling Technology |
| **Phosphorylated FOXO3a (S413)** | #8174S | 1:1000 | Cell Signaling Technology |
| **FOXO3a** | #2494S | 1:2000 | Cell Signaling Technology |
| **Gp130** | #3732S | 1:1000 | Cell Signaling Technology |
| **Puromycin Incorporation** | #MABE343 | 1:4000 | Millipore |
| **MyHC-Fast** | #M4276 | 1:4000 | Sigma |
| **MyHC-Slow** | #M8421 | 1:4000 | Sigma |
| **Secondary anti-rabbit** | (#7074) | 1:4000 | Cell Signaling Technology |
| **Secondary anti-mouse** | (#7076) | 1:4000 | Cell Signaling Technology |


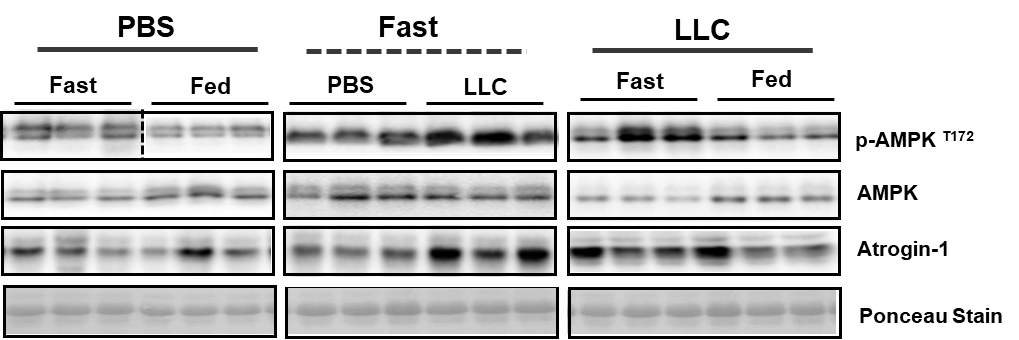

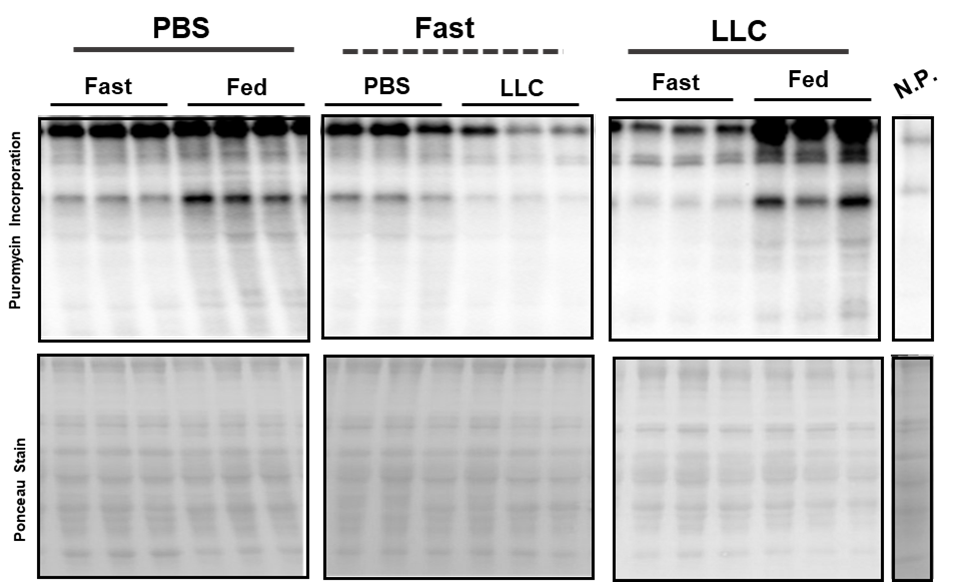

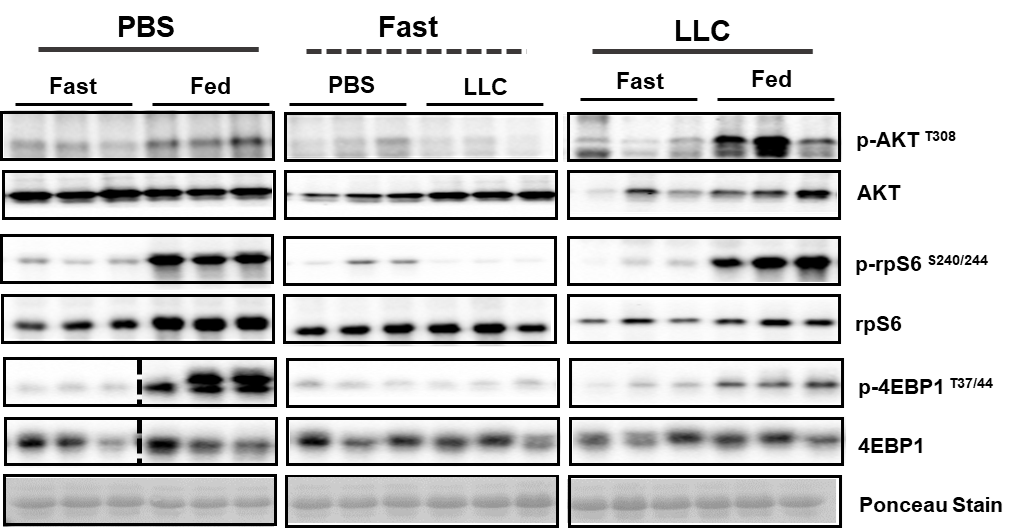


**C.**

Legend: Representative western blotting images of A) phosphorylated (p-) to total AKT^(T308)^, rpS6^(S240/244)^, and 4EBP1^(T37/44)^ ; B) puromycin incorporation; C) phosphorylated (p-) to total AMPK^(T172)^ and atrogein-1 protein expression with Ponceau S stain as a protein loading control. Dashed lines represents different areas of the same gel.

**B.**

**A.**

**Supplement Figure 1: Cachexia in LLC tumor-bearing mice.**


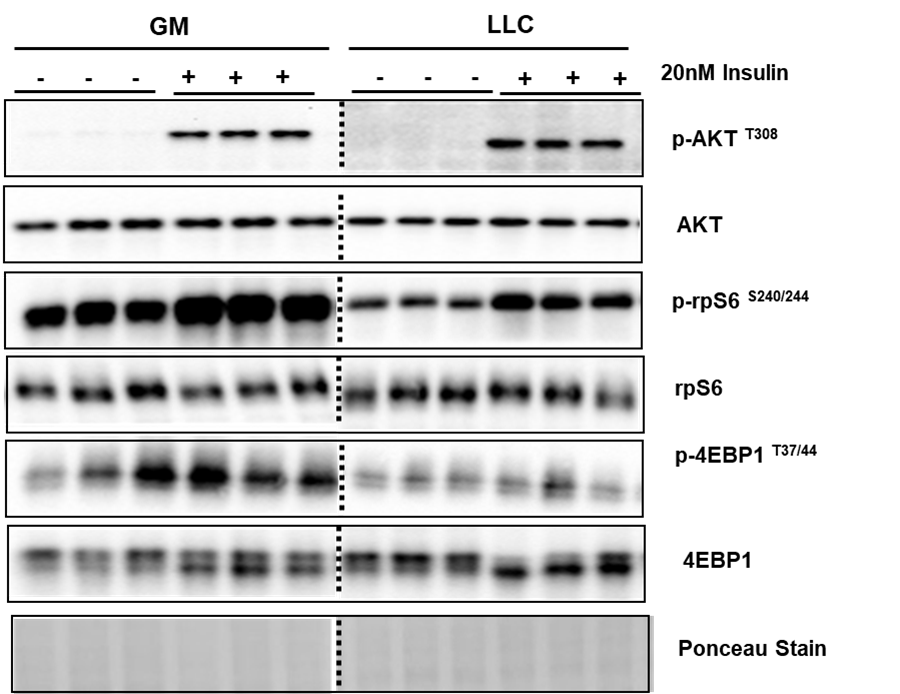

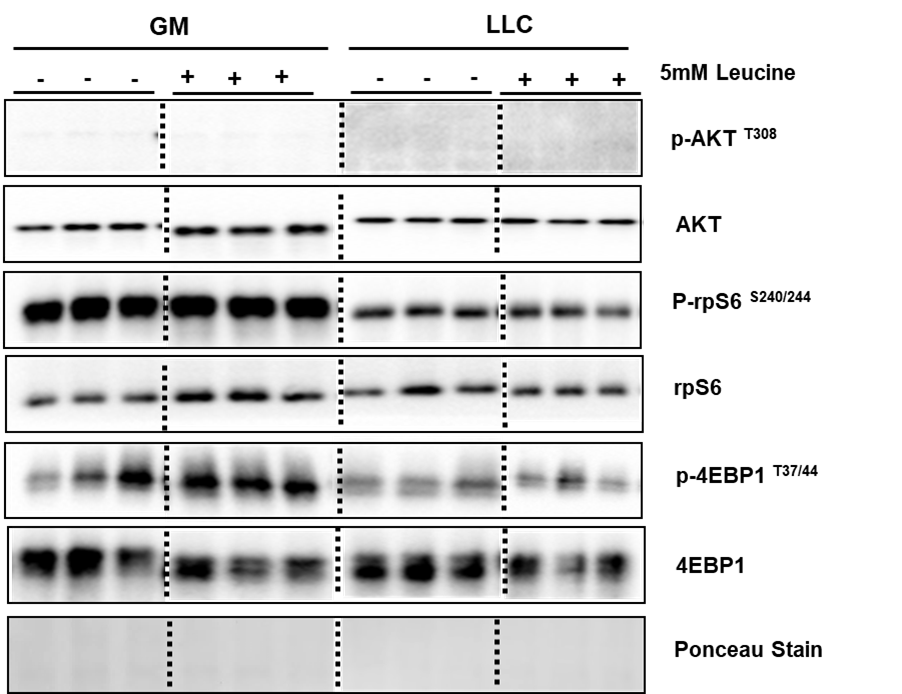

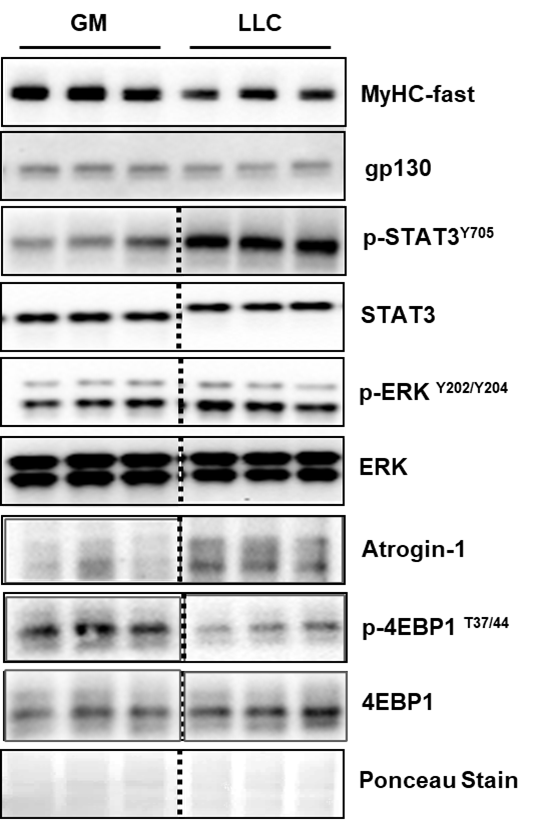


Legend: Representative western blotting images of A) Myhc-fast, gp130, atrogin-1, phosphorylated (p-) to total AKT^(T308)^, rpS6^(S240/244)^, and 4EBP1^(T37/44)^ ; B) phosphorylated (p-) to total AKT^(T308)^, rpS6^(S240/244)^, and 4EBP1^(T37/44)^ ; C) phosphorylated (p-) to total AKT^(T308)^, rpS6^(S240/244)^, and 4EBP1^(T37/44)^protein expression with Ponceau S stain as a protein loading control. Dashed lines represents different areas of the same gel.

**C.**

**B.**

**A.**

**Supplement Figure 2: C2C12 myotubes treated with LLC conditioned media alters the mTORC1 response to insulin and leucine**


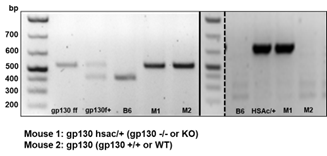

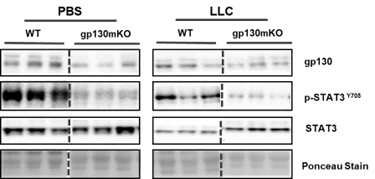

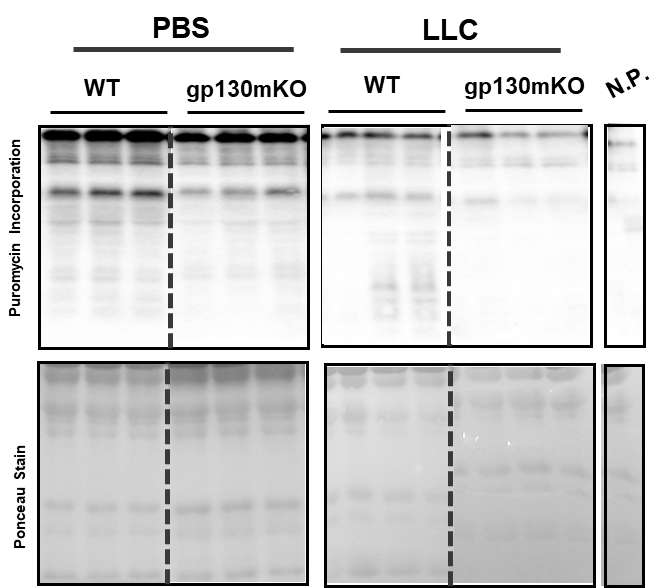

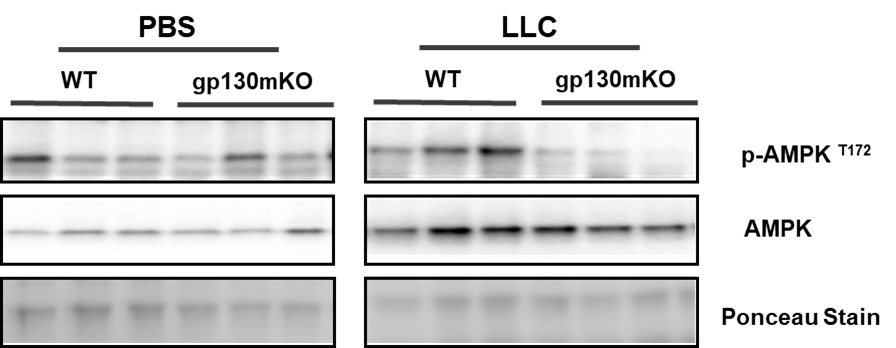

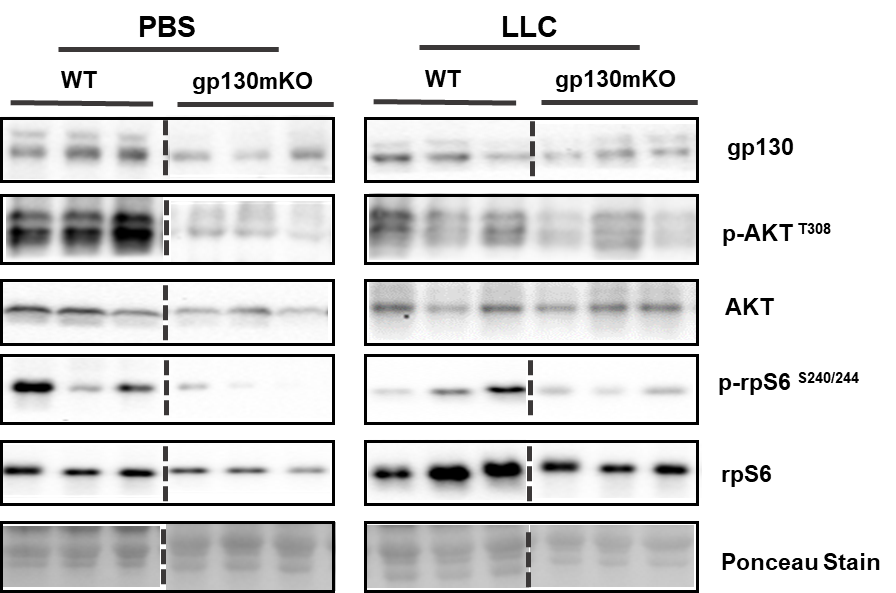


Legend: A) Example of tail snip confirmation of gp130 floxed  and gp130 floxed HSA-MCM genotypes. Representative western blotting images of A) gp130 and  phosphorylated (p-) to total STAT3^(Y705)^ ; B)phosphorylated (p-) to total AKT^(T308)^ and rpS6^(S240/244)^;  C) puromycin incorporation; D) phosphorylated (p-) to total AMPK^(T172)^ protein expression with Ponceau S stain as a protein loading control. Dashed lines represents different areas of the same gel.

**Supplement Figure 3: Cachexia in gp130 LLC tumor-bearing mice.**

**A.**

**B.**

**D.**

**E.**

**C.**


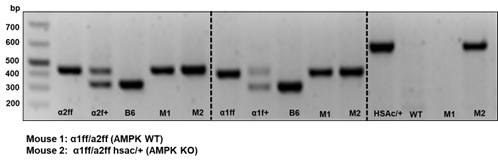

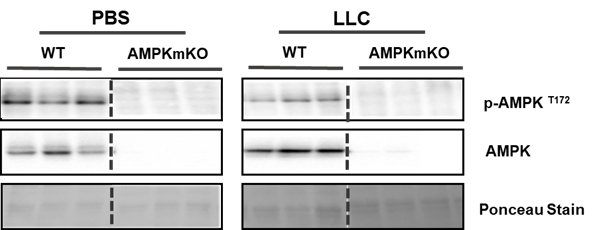

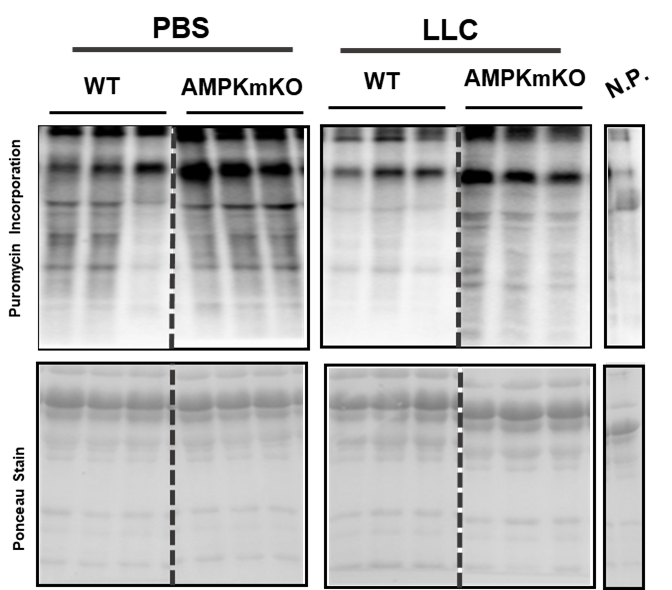

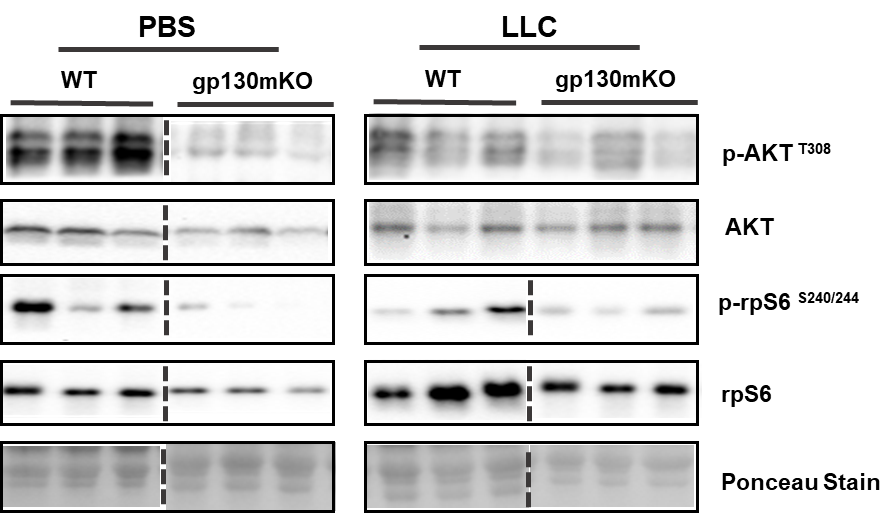


Legend: A) Example of tail snip confirmation of AMPKa***^1^***a***^2^*** floxed and AMPKa***^1^***a***^2^*** floxed HSA-MCM genotypes. Representative western blotting images of A) phosphorylated (p-) and total AMPK^(T172)^; B) phosphorylated (p-) to total AKT^(T308)^ and rpS6^(S240/244)^; C) puromycin incorporation with Ponceau S stain as a protein loading control. Dashed lines represents different areas of the same gel.

**Supplement Figure 4: Cachexia in AMPK LLC tumor bearing mice.**

**A.**

**B.**

**C.**

**D.**
